# Active migration technique in RIRS for 1‐ to 2‐cm middle and upper ureteral stones in a prospective randomized controlled study

**DOI:** 10.1002/bco2.70204

**Published:** 2026-04-21

**Authors:** Ping Liang, Qing‐lai Tang, Juan‐juan Mao, Yu‐xin Zhang, Yun‐peng Li, Xing‐zhu Zhou, Rong‐zhen Tao

**Affiliations:** ^1^ From the Department of Urology The second Hospital of Nanjing, Affiliated to Nanjing University of Chinese Medicine Nanjing Jiangsu China; ^2^ From the Department of Urology The Affiliated Jiangning Hospital of Nanjing Medical University Nanjing Jiangsu China

**Keywords:** active migration technique, flexible and negative suction ureteral access sheath, retrograde intrarenal surgery, stone‐free rates, ureteral stones

## Abstract

**Objective:**

This study aimed to observe the efficacy and safety of the active migration technique and in situ lithotripsy technique in retrograde intrarenal surgery (RIRS) for patients with 1‐ to 2‐cm middle and upper ureteral stones.

**Patients and Methods:**

Two hundred seven patients were enrolled in the study, of which 103 included in the study group received active migration lithotripsy and 104 included in the control group received in situ lithotripsy. The primary study outcome was the stone‐free rate (SFR) on the first postoperative day. Secondary outcomes included the total SFR 4 weeks postoperatively, operative time, reduction in the haemoglobin levels, the length of postoperative hospital stay, the incidence of ureteral stricture at 3‐month postoperatively and any surgery‐related complications.

**Results:**

There was no obvious difference between two groups in patients' demographics and preoperative clinical characteristics (*p* > 0.05). The operative time was significantly shorter in the study group than in the control group (57.1 vs. 62.5 min, *p* < 0.001). The study group also had significantly higher immediate and total SFRs (81.5% vs. 64.4%, *p* = 0.006, 90.3% vs. 77.9%, *p* = 0.015, respectively). At 3 months postoperatively, the incidence of ureteral stricture in the study was statistically lower than in the control group (1.0% vs. 6.7%, *p* = 0.032). Notablely, the overall complication rate was significantly lower in the study group than in the control group (*p* < 0.001).

**Conclusions:**

Our study provides evidence that the active migration technique, when combined with flexible and negative suction ureteral access sheath (FANS) in RIRS, results in a higher SFR and a lower complication rate than in situ lithotripsy for treating 1‐ to 2‐cm middle and upper ureteral stones. The protocol for this study has been accepted by the Chinese Clinical Trial Registry (The registration number: ChiCTR2200056402; Date of registration: 03‐06‐2022).

## 1 INTRODUCTION

Ureteral stones are a common condition in urology. Compared with lower ureteral stones, the treatment of middle and upper ureteral stones is often more challenging.^1^ Among the various treatment options for ureteral stones measuring 1–2 cm, retrograde intrarenal surgery (RIRS) has become a widely adopted approach due to its minimally invasive nature compared with percutaneous nephrolithotomy (PCNL) and its greater versatility compared with extracorporeal shock wave lithotripsy (ESWL).^2^ In line with this, the European Association of Urology (EAU) recommends RIRS as the primary treatment option for ureteral stones ≥1 cm.^3^

Most clinicians are accustomed to using ureteroscopy for in situ lithotripsy. However, due to the narrow lumen and natural curvature of the ureter, the laser fibre often cannot make direct contact with the stone, requiring repeated adjustments in angle to achieve lithotripsy.^4^ This not only increases the complexity and duration of the procedure but also easily elevates the risk of ureteral injury. Reported stone‐free rates (SFRs) for 1‐ to 2‐cm middle and upper ureteral stones treated with ureteroscopy vary widely (45.6%–96.7%), largely depending on the surgical strategy employed.^5^ The recent introduction of the flexible and negative suction ureteral access sheath (FANS) in RIRS represents a significant advancement. Studies have shown that combining FANS with RIRS can significantly improve SFR and reduce postoperative complications in patients with upper urinary tract stones.^6^

In our department, we have optimized the treatment strategy for middle and upper ureteral stones by initially pushing the stones into favourable renal calyces—typically the upper and middle calyces—before performing laser lithotripsy. We refer to this approach as the active migration technique. We hypothesize that its advantages include (1) reducing the need for lithotripsy within the ureter, thereby minimizing ureteral damage; and (2) fragmenting stones in a semienclosed space, which prevents stone fragments from dispersing throughout the renal pelvis and potentially improves the SFR. To date, no studies have evaluated the use of the active migration technique in combination with FANS for treating middle and upper ureteral stones. Therefore, we conducted a prospective, randomized controlled trial to assess and compare the efficacy and safety of the active migration technique versus the conventional in situ lithotripsy technique in RIRS for patients with 1‐ to 2‐cm middle and upper ureteral stones.

## 2 MATERIALS AND METHODS

### 2.1 Study design and patients

**FIGURE 1:**
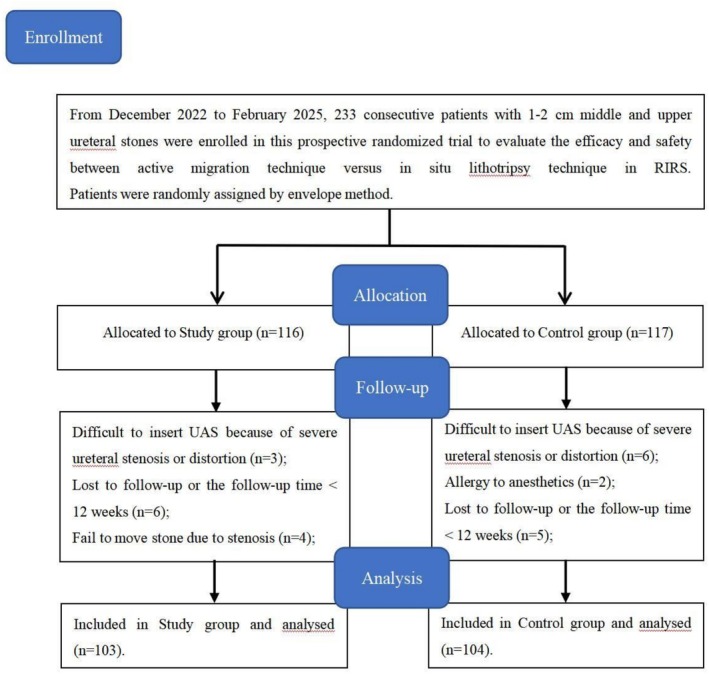
Flowchart for cases selection of the trial outlining enrollment, randomization, allocation, follow‐up and analysis according to intention‐to‐treat standards. RIRS, retrograde intrarenal surgery.

Patients with 1‐ to 2‐cm middle and upper ureteral stones who were referred to our institute were considered for this prospective, randomized controlled study conducted between December 2022 and February 2025. After applying strict inclusion and exclusion criteria, as outlined in Table 1, the patients were randomly assigned to two groups by using the envelope method. Finally, 207 patients were enrolled in the study, of which 103 included in the study group received active migration lithotripsy and 104 included in the control group received in situ lithotripsy, which was decided based on power analysis performed to estimate the sample size (Figure 1). The participants' pretreatment evaluation included medical history, physical examination, laboratory investigations (i.e., urine analysis, urine culture and/or sensitivity, complete blood count, blood urea nitrogen and the serum levels of creatinine, C‐reactive protein and procalcitonin) and radiological investigations. Patients with a known urinary tract infection (UTI) received antibiotic treatment until the infection was under control. The study was approved by the clinical research ethics committee of the Affiliated Jiangning Hospital of Nanjing Medical University (ethics approval number: 2025‐03‐132‐K01). Written informed consent was obtained from all participants. The study followed the principles of the Helsinki Declaration.

**TABLE 1:** The inclusion criteria and exclusion criteria in the study.

The inclusion criteria	The exclusive criteria
Patients' age was 18 to 70 years	Uncontrollable UTI and requires drainage
Diagnosed as 1.0–2.0 cm single middle or upper ureteral stones confirmed by CT	Severe cardiovascular and cerebrovascular diseases
No contraindications for surgery	Pregnancy or coagulation disorders
The time from diagnosis to surgery was less than 1 month	Combining ipsilateral renal stones or bilateral ureteral stones required a one‐stage surgery
Ability to provide written informed consent and comply with the trial requirements	History of ureteral stenosis or impacted ureteral stones evaluating through preoperative imaging examination^7^
American Society of Anesthesiology score 1–3	Unable to understand or comply with trial records

### 2.2 Randomization and masking

Parallel randomization was conducted by using a stratified approach in our study. Our centre enrolled 207 participants, who were then randomized in a 1:1 ratio to either the study or the control group. The randomization sequence was arranged electronically before patient inclusion. Consecutively numbered and sealed envelopes were used for random sequence allocation and concealment. After subjecting the patients to general anaesthesia and before ureteroscopy was entered into the urethra, the sealed envelope was opened by a designated nurse to reveal the specific surgical approach to be undertaken. Subsequently, after the procedure, the same nurse automatically recorded the operative data.

### 2.3 Perioperative and surgical procedures

All patients underwent preoperative imaging, including plain abdominal radiography of the kidneys, ureters and bladder (KUB) and noncontrast CT to evaluate hydronephrosis, as well as to assess the size, location, number and specific details related to the ureteral stones. Accordingly, preprocedural urine cultures were prepared and used in appropriate antibiotic therapy as per the results of the culture‐antibiogram test. Patients showing negative urine cultures were treated with broad‐spectrum antibiotics before the surgery (i.e., intravenous cefuroxime 1.5 g or levofloxacin 500 mg, if allergic). Otherwise, the procedures were scheduled once the infection indicators displayed a downward trend after the application of sensitive antibiotics (mainly intravenous piperacillin sodium and tazobactam sodium) and after confirming a negative urine culture. The stone size was defined as the largest diameter of a single stone on preoperative KUB and/or noncontrast CT. All procedures were conducted by two urologists, each with experience in conducting more than 200 RIRS procedures annually. The surgical method for the enrolled patients was randomly selected, thereby excluding any subjective bias.

#### 2.3.1 Study group procedure

**FIGURE 2:**
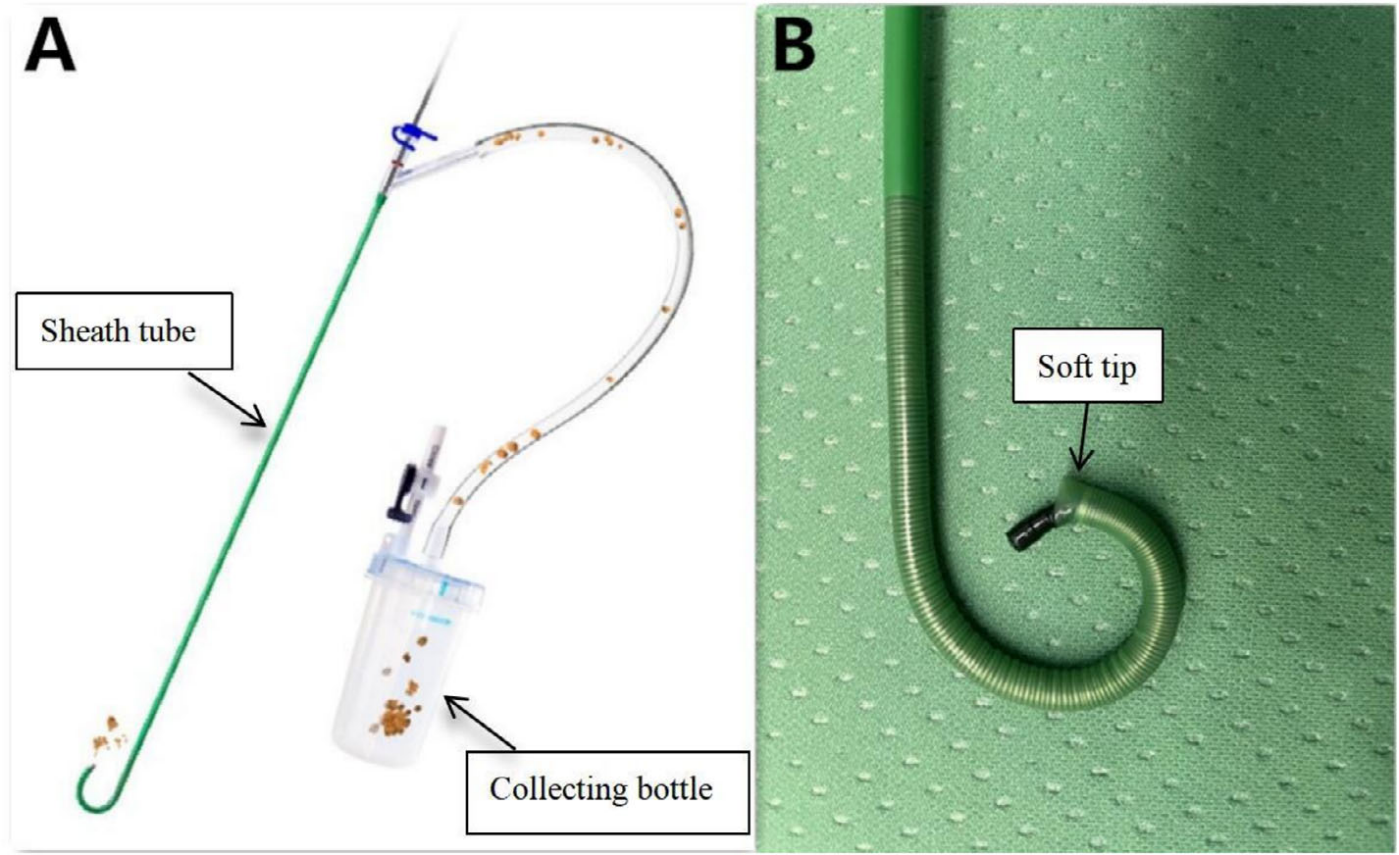
Structural diagrams of the flexible and negative suction ureteral access sheath (FANS). (A) Whole view of the FANS; (B) diagram of the proximal end of the FANS.

**FIGURE 3:**
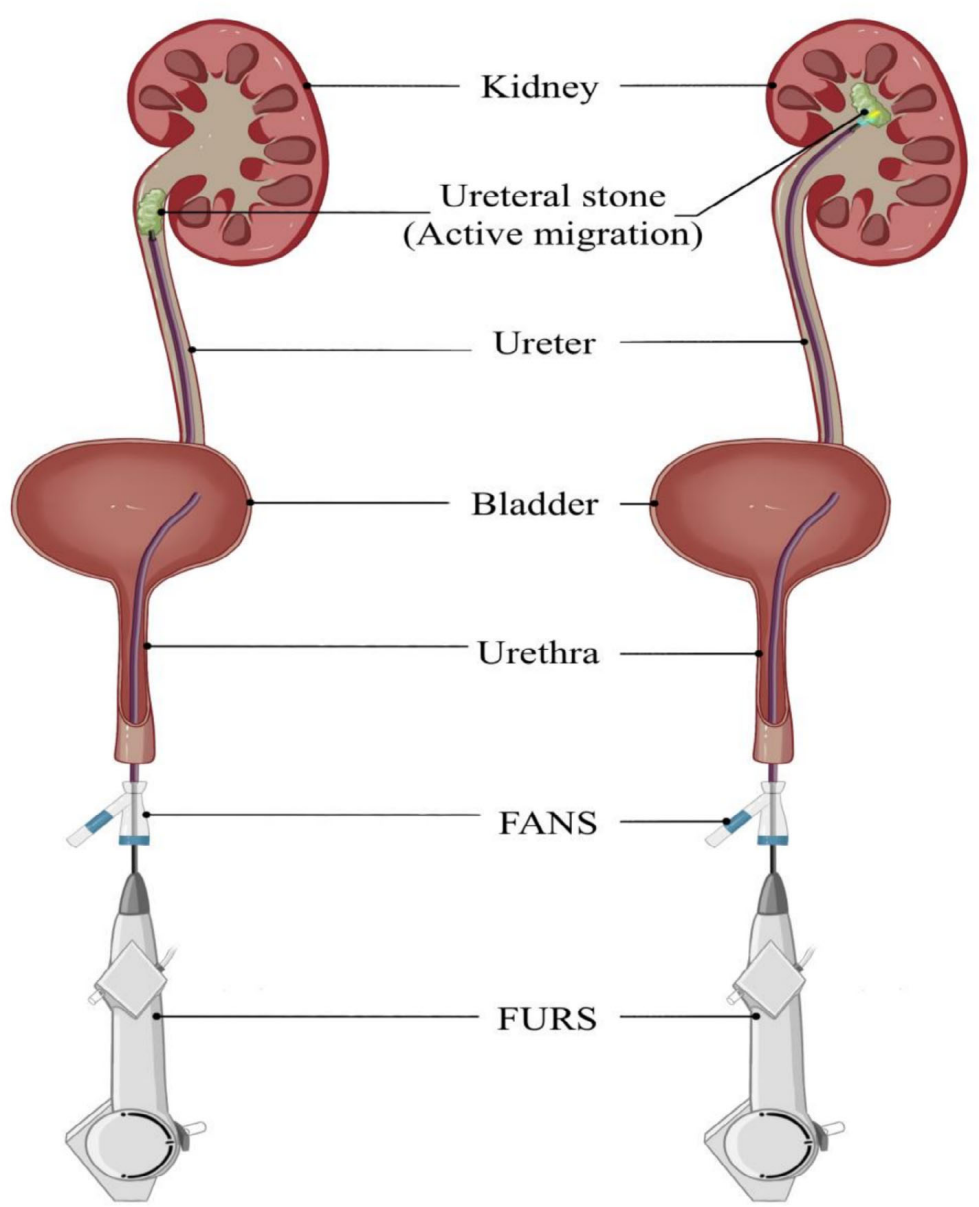
Simulation diagrams of the active migration technique combined with the flexible and negative suction ureteral access sheath (FANS) in retrograde intrarenal surgery (RIRS).

Under general anaesthesia, patients were placed in the Trendelenburg lithotomy position (head down by 30°) for retrograde endoscopic access. A ureteroscopy (STORZ, 8/9.8‐Fr) was performed to identify the location of stone obstruction, and the stone was pushed back into the renal pelvis under water flow, the tip of the ureteroscope or guidewire (Bard, USA). Then, a loach guidewire was introduced to access the upper urinary tract, followed by the placement of a 12/14‐Fr or 11/13‐Fr FANS (length: 40 cm for females; 50 cm for males) (Wellead Medical, Guangdong, China) into the upper affected ureter (Figure 2). The advantage offered by this sheath was that the 3‐mm soft tip was designed without a metal spring coil, which provided optimal protection for the ureter mucosa.^7^ Occurrence of ureteral stenosis or distortion during surgery could lead to the failure of FANS implantation; therefore, balloon dilation was attempted as the first line of approach. In cases not feasible for dilation, only a double‐J stent was inserted for ureteral expansion. A 7.5‐Fr disposable electronic flexible ureteroscope (fURS) (Pusen Medical, Guangdong, China) was then inserted through the FANS, and the FANS was adjusted to encase the stone within the renal collecting system (Figure 3). We usually set the fluid irrigation flow to 80–100 mL/min and a negative pressure suction to 85–90 mmHg to obtain a clear surgical view. The lithotripsy process was performed using a holmium laser with a 200‐μm laser fibre under an energy of (0.6–0.8 J) * (20–30 Hz). Small fragments were automatically aspirated through the gap between the fURS and FANS, whereas larger fragments required the fURS to be repeatedly inserted and withdrawn slowly under continuous suction from the body (Video S1). At the end of the procedure, the fURS was directed toward the collecting system to retrieve any remaining large stone fragments. The FANS and fURS were removed under direct visualization to document and evaluate any ureteral injury.^8^ A 6‐Fr double‐J stent (Bard, USA) was placed in all patients postoperatively.

#### 2.3.2 Control group procedure

General anaesthesia was administered, and the lithotomy position was used for each patient in this group. A ureteroscopy was performed to retrogradely access the ureter until reaching the stone, and the surroundings of the stone were observed. We usually place a stone occlusion device (IVX‐SC10; Innovex Medical, Shanghai) along the gap between the stone and the ureteral mucosa above the stone. Presently, the stone was fragmented with a holmium laser using a 200‐μm laser fibre (with energy setting <15 W). Then, the stone fragments were removed using a nitinol stone basket (Cook Medical, USA). Similarly, ureteroscopy was performed to examine the entire ureter, and a 6‐Fr double‐J stent was indwelt.

### 2.4 Postoperative follow‐up

The level of white blood cell counts, C‐reactive protein, and procalcitonin at 2‐h postoperatively was monitored to screen out serious UTI. Then, 1‐mm‐thick sections from all patients were subjected to ultralow‐dose, noncontrast CT scanning on the first postoperative day and then at 4 weeks after the surgery to evaluate the immediate and total SFR. Two groups of patients were discharged within 48 h of the surgery if they did not experience any significant discomfort. Every patient received health education before discharge, including recommendations related to precautions, follow‐up dates and emergency contact information. Double‐J stents were removed within 4 weeks of the surgery. Meanwhile, the stone composition analyses were performed for all patients to obtain a reference for metabolic analysis and subsequent prevention.

Stone‐free status was defined as the complete absence of residual stone fragments or the presence of clinically insignificant fragments measuring ≤2 mm, asymptomatic, non‐obstructive and noninfectious.^9^ The primary study outcome was the SFR on the first postoperative day. The secondary outcomes included the total SFR 4 weeks postoperatively (evaluated through ultralow‐dose CT), operative time, reduction in the haemoglobin levels, the length of postoperative hospital stay, the incidence of ureteral stricture at 3‐month postoperatively and any surgery‐related complications.

The operative time was calculated from the time since the ureteroscope was inserted into the urethra up to the time when the double‐J tube was placed. Reduction in the haemoglobin levels was deemed indicative of the difference between preoperative haemoglobin levels and the 2‐h postoperative haemoglobin level. The length of postoperative hospital stay was counted from the day of surgery to the time of discharge. Importantly, postoperative hydronephrosis was monitored, particularly for ureteral stone patients. Regular urinary ultrasound examinations were conducted for all patients on a monthly basis. Patients were asked to undertake IVU or enhanced CT and provided a follow‐up treatment plan if their hydronephrosis had significantly worsened relative to their preoperative imaging at 3 months postoperatively. Postoperative complications were classified using the modified Clavien grading system, including fever (≥38.5°C), lower back pain, perirenal hematoma, blood transfusion and urosepsis.^10^ For patients with residual stones, additional auxiliary procedures were performed at least 4 weeks after the surgery, including external physical vibration lithotripsy, ESWL or positional therapy, as deemed appropriate.^11^, ^12^

### 2.5 Statistical analysis

Statistical analysis was conducted using IBM SPSS Statistics for Windows, Version 22.0 (IBM Corp., Armonk, NY, USA). Continuous variables were reported as the means ± standard deviations. The independent samples *t*‐test was performed to compare the patient demographics, follow‐up data and surgical outcomes between the groups, whereas the Shapiro–Wilk test was performed to assess the normality of the initial data. For categorical variables, including other pre‐ and postoperative clinical characteristics, comparisons were made using the chi‐squared test. *p* < 0.05 was considered to indicate statistical significance.

## 3 RESULTS

### 3.1 Demographics and preoperative clinical characteristics

In total, 207 patients were randomly assigned to either the study group (*n* = 103) or the control group (*n* = 104). No significant differences in baseline demographics or preoperative clinical characteristics were noted between the two groups (Table 2). The mean stone size was 1.5 cm in the study group and 1.6 cm in the control group. Both groups were also comparable in terms of age, body mass index, sex ratio, history of hypertension or diabetes, ASA classification, stone laterality, stone composition, hydronephrosis grade, urine culture results and history of previous upper urinary stone surgeries (all *p* > 0.05).

**TABLE 2:** Comparisons of patients' demographics and preoperative clinical characteristics between two groups.

Variables, mean ± SD or *n* (%)	Study group (*n* = 103)	Control group (*n* = 104)	*t*/*χ* ^2^ value	*p*‐Value
**Age, years**	53.2 ± 4.3	54.1 ± 4.1	−1.541	0.125
**BMI, kg/m** ^**2**^	24.5 ± 3.3	23.9 ± 3.5	1.269	0.206
**Gender**			0.416	0.519
Male	62 (60.2)	58 (55.8)	‐	‐
Female	41 (39.8)	46 (44.2)	‐	‐
**Hypertension history**	43 (41.7)	48 (44.4)	0.408	0.523
**Diabetes history**	35 (34.0)	29 (27.9)	0.901	0.343
**ASA classification**			1.681	0.711
I	34 (33.0)	29 (27.9)	‐	‐
II	57 (55.3)	61 (58.6)	‐	‐
III	12 (11.7)	14 (13.5)	‐	‐
**Laterality**			0.826	0.363
Left	59 (57.3)	66 (63.5)	‐	‐
Right	44 (42.7)	38 (36.5)	‐	‐
**Stone diameter (cm)**	1.5 ± 0.5	1.6 ± 0.4	−1.589	0.114
**CT value of stone (HU)**	1017.5 ± 124.1	996.3 ± 114.9	1.275	0.204
**Grade of hydronephrosis**			0.642	0.423
None or mild	66 (64.1)	61 (58.7)	‐	‐
Moderate or severe	37 (35.9)	43 (41.3)	‐	‐
**Urine culture**			0.166	0.684
Negative	73 (70.9)	71 (68.3)	‐	‐
Positive	30 (29.1)	33 (31.7)	‐	‐
**Prestenting**	39 (37.9)	35 (33.6)	0.399	0.527
**Upper urinary stone operation histories** ^**a**^	22 (21.3)	27 (26.0)	0.607	0.436

Abbreviations: ASA, American Society of Anesthesiologists; BMI, body mass index; CT, computed tomography; SD, standard deviation.

^a^ Upper urinary stone operation histories include flexible ureteroscope lithotripsy, percutaneous nephrolithotomy or open surgery for stone.

### 3.2 Postoperative clinical characteristics

Postoperative clinical outcomes are summarized in Table 3. The mean decrease in haemoglobin and length of postoperative hospital stay did not significantly differ between the two groups (both *p* > 0.05). However, the operative time was significantly shorter in the study group than in the control group (57.1 vs. 62.5 min, *p* < 0.001). The study group also had significantly higher immediate and total SFRs (81.5% vs. 64.4%, *p* = 0.006, 90.3% vs. 77.9%, *p* = 0.015, respectively). At 3 months postoperatively, ureteral stricture was observed in seven patients in the control group compared with one patient in the study group—a statistically significant difference (6.7% vs. 1.0%, *p* = 0.032).

**TABLE 3:** Comparisons of surgical outcomes and postoperative clinical characteristics between two groups.

Variables, mean ± SD or *n* (%)	Study group (*n* = 103)	Control group (*n* = 104)	*t*/*χ* ^2^ value	*p*‐Value
**Immediate SFR**	84 (81.5)	67 (64.4)	7.695	0.006**
**Total SFR**	93 (90.3)	81 (77.9)	5.944	0.015*
**Operative time, min**	57.1 ± 4.3	62.5 ± 4.9	−8.424	< 0.001**
**Haemoglobin decrease, g/L**	7.2 ± 2.4	7.7 ± 2.1	−1.596	0.112
**Postoperative hospital stays, days**	1.5 ± 0.3	1.6 ± 0.6	−1.514	0.132
**3 months ureteral stricture**	1 (1.0)	7 (6.7)	4.621	0.032*
**Clavien–Dindo**			12.121	< 0.001**
Grades I–II	5 (4.8)	17 (16.3)	‐	‐
Grades III–IV	0 (0.0)	5 (4.8)	‐	‐
**Complications**				
Fever (>38.5°C) (Clavien Grade I)	3 (2.9)	11 (10.6)	4.821	0.028*
Low back pain (Clavien Grade I)	4 (3.9)	14 (13.5)	5.979	0.014*
Perirenal hematoma (Clavien Grade II)	1 (1.3)	8 (7.7)	5.622	0.018*
Blood transfusion (Clavien Grade II)	0 (0.0)	2 (1.9)	2.000	0.157
Urosepsis (Clavien Grade IV)	0 (0.0)	5 (4.8)	5.075	0.024*
**Stone compositions**			0.544	0.909
Calcium oxalate	52 (50.5)	55 (52.9)	‐	‐
Calcium phosphate	15 (14.6)	13 (12.5)	‐	‐
Struvite or carbonated apatite	27 (26.2)	29 (27.9)	‐	‐
Uric acid or cysteine	9 (8.7)	7 (6.7)	‐	‐

Abbreviations: QoL, quality of life; SD, standard deviation; SFR, stone‐free rate.

* *p* < 0.05.

** *p* < 0.01.

Regarding postoperative safety, the overall complication rate was significantly lower in the study group than in the control group (*p* < 0.001). Specific complications such as fever >38.5°C (2.9% vs. 10.6%, *p* = 0.028), lower back pain (3.9% vs. 13.5%, *p* = 0.014), perirenal hematoma (1.3% vs. 7.7%, *p* = 0.018) and urosepsis (0% vs. 4.8%, *p* = 0.024) were significantly more frequent in the control group. Blood transfusions were required in two patients in the control group, but in none in the study group, although this difference between the two groups was not statistically significant (*p* > 0.05). Postoperative stone composition analysis revealed no significant differences between the two groups (*p* > 0.05).

## 4 DISCUSSION

The retrograde migration of middle and upper ureteral stones during lithotripsy is considered one of the primary factors negatively affecting the SFR in ureteroscopy. The migration of larger stone fragments into blind spots, especially the lower calyces, can reduce SFR and often necessitates additional treatment.^13^ Based on our experience, the primary causes of stone displacement include the retrograde flow of intraoperative perfusion fluid during surgery and the pulse effect of the laser. To address this issue, doctors have adopted various strategies. One of the most commonly used methods is the placement of a stone occlusion device above the ureteral stone. Yi X et al. reported that ureterolithotripsy combined with a stone occlusion device for in situ lithotripsy was associated with low rates of stone migration, minimal complications and high SFR.^14^ Additionally, patient positioning may significantly influence stone migration during lithotripsy. Previous prospective, randomized, comparative studies have shown that the reverse Trendelenburg position is a safe and effective surgical method for treating proximal ureteral stones, offering reduced stone migration, higher SFR, shorter operative times and fewer postoperative complications.^15^, ^16^ Despite these strategies, in situ lithotripsy within the ureter still has notable drawbacks.^17^, ^18^ First, due to the narrow ureteral lumen, laser lithotripsy can easily damage the surrounding mucosa, increasing the risk of ureteral stricture. Second, in cases involving a bent ureter, the angle for stone fragmentation is severely limited, making the procedure more technically challenging and time‐consuming. Lastly, stone fragments migrating into the renal pelvis during lithotripsy can contribute to a low SFR. Therefore, to further explore the efficacy and safety of in situ lithotripsy versus active migration lithotripsy, we conducted this prospective clinical study.

Efficacy is the first issue that clinical research must address. Based on the aforementioned considerations, we developed a specific operative strategy. A key component of our approach is active stone migration. We always aim to mobilize the stone into the renal pelvis using the irrigation flow, the tip of the ureteroscope or a guidewire. A recent meta‐analysis reported an overall ureteral stricture rate of 1.9% following ureteroscopy, rising to 2.7% in studies from the past 5 years and up to 4.9% when stones were impacted.^19^ The choice of treatment method is likely the main contributing factor. Our study sought to minimize ureteral trauma by avoiding laser lithotripsy within the ureter itself. Accordingly, at 3 months postoperatively, the ureteral stricture rate in the study group was significantly lower than that in the control group (1.0% vs. 6.7%, *p* = 0.032).

Then, patients in the study group were placed in the Trendelenburg lithotomy position (head down 30°) to facilitate retrograde access and promote stone migration into the upper or middle calyces—areas considered optimal for RIRS. Operating in the renal lower calyx was avoided whenever possible. Fragmenting stones in the renal calyces helps to restrict stone movement, enhances fragmentation efficiency and improves stone removal. Consequently, our findings demonstrated that the study group had significantly higher immediate and total SFRs than the control group (81.5% vs. 64.4%, *p* = 0.006; 90.3% vs. 77.9%, *p* = 0.015, respectively). In addition, with the support of FANS during RIRS, the study group benefited from a clearer surgical field. The balance between irrigation and negative pressure allowed for simultaneous lithotripsy and suction. Consequently, the operative time was significantly shorter in the study group than in the control group (57.1 vs. 62.5 min, *p* < 0.001).

Safety is another critical aspect in clinical research. UTI is one of the most frequent complications following RIRS, with reported incidence ranging from 1.7% to 18.8%.^20^ A major contributing factor is elevated intrarenal pressure (IRP) during surgery. Some studies have shown that applying negative pressure technology in RIRS can reduce infection rates by lowering IRP.^21^, ^22^ FANS addresses this issue effectively by passively bending with the fURS to traverse the ureteropelvic junction and reach the renal pelvis and calyces. Our study confirmed that the incidences of fever (>38.5°C), lower back pain, perirenal hematoma and urosepsis were significantly lower in the study group than in the control group (2.9% vs. 10.6%, *p* = 0.028; 3.9% vs. 13.5%, *p* = 0.014; 1.3% vs. 7.7%, *p* = 0.018, 0% vs. 4.8%, *p* = 0.024, respectively).

Despite promising results, this study has several limitations. Firstly, the 3‐month follow‐up period may not be sufficient to detect long‐term complications such as delayed ureteral strictures. Secondly, the study did not include a comprehensive cost‐effective analysis, which would have provided valuable insights into the economic implications of FANS use compared with conventional ureteroscopic lithotripsy. Thirdly, as stone fragmentation was intentionally performed in different anatomical environments between the two groups, this factor may have influenced operative efficiency and outcomes. Lastly, as a single‐centre study with a modest sample size, the potential for sampling bias exists. Optimal procedures will likely emerge from extended clinical applications and observations over time.

## 5 CONCLUSIONS

Our study provides evidence that the active migration technique, when combined with FANS in RIRS, results in a higher SFR and a lower complication rate than in situ lithotripsy for treating 1‐ to 2‐cm middle and upper ureteral stones. This technique is safe, effective and reproducible in clinical practice. However, further validation through large‐scale multicenter prospective studies is necessary to substantiate the aforementioned findings.

## AUTHOR CONTRIBUTIONS

**Qing‐lai Tang and Rong‐zhen Tao:** Project development. **Ping Liang and Yu‐xin Zhou:** Data collection. **Yun‐peng Li, Juan‐juan Mao and Yu‐xin Zhang:** Data analysis and manuscript writing.

## CONFLICT OF INTEREST STATEMENT

The authors declare no conflicts of interest.

## Supporting information


**Video S1.** Supporting Information.

## Data Availability

The datasets used and analysed during the current study are available from the corresponding author on reasonable request.
